# Association of sun-seeking behaviors with indoor tanning behavior in US white females during high school/college in Nurses’ Health Study II

**DOI:** 10.1186/s12889-024-17716-6

**Published:** 2024-01-11

**Authors:** Bojung Seo, Sheng Yang, Eunyoung Cho, Abrar A Qureshi, Jiali Han

**Affiliations:** 1 Department of Epidemiology, Richard M. Fairbanks School of Public Health, Indiana University, 1050 Wishard Blvd, Health Sciences Building, RG 6124, Indianapolis, IN 46202 USA; 2 Department of Dermatology, Alpert Medical School of Brown University, Providence, RI USA; 3 Department of Epidemiology, Brown University School of Public Health, Providence, RI USA; 4 Indiana University Melvin and Bren Simon Cancer Center, Indianapolis, IN USA; 5 Channing Division of Network Medicine, Department of Medicine, Brigham and Women’s Hospital, Boston, USA

**Keywords:** Sun exposure, Tanning bed, Sun-seeking, Skin cancer, Tanning behavior

## Abstract

**Background:**

Frequent exposure to ultraviolet light has more detrimental and longer-term effects on the skin in early life than in adulthood. Teenagers with strong sun-seeking behaviors may be more likely to use an indoor tanning bed than those who seek less sun. We aimed to examine associations between sun-seeking behaviors and indoor tanning behavior during high school/college in US females.

**Methods:**

In this cross-sectional study, we used data from The Nurses’ Health Study II, a large prospective cohort of US female nurses. We included a total of 81,746 white females who provided responses on the average annual frequency of indoor tanning during high school/college. Our study exposures were number of times/week spent outdoors in a swimsuit and percentage of time wearing sunscreen at the pool/beach as a teenager, weekly hours spent outdoors in direct sunlight during the daytime during high school/college, and number of severe sunburns that blistered between ages 15–20 years. The main outcome was annual frequency of indoor tanning bed usage during high school/college.

**Results:**

In multivariable-adjusted logistic regression, we demonstrated positive associations between sun-seeking behaviors and indoor tanning use. Specifically, teenagers who spent 7 times/week outdoors in a swimsuit (adjusted odds ratio [aOR], 95% confidence interval [CI] for daily vs. <1/week: 2.68, 1.76–4.09) were more likely to use indoor tanning beds ≥ 12 times/year. Teenagers with ≥ 10 sunburns (aOR, 95% CI for ≥ 10 vs. never: 2.18, 1.53–3.10) were more likely to use indoor tanning beds ≥ 12 times/year. Also, teenagers/undergraduates who spent ≥ 5 h/week outdoors in direct sunlight (aOR, 95% CI for ≥ 5 h/week vs. <1 h/week: 2.18, 1.39–3.44) were more likely to use indoor tanning ≥ 12 times/year. However, there was not a significant association between average usage of sunscreen at the pool/beach and average usage of indoor tanning beds. Multivariable-adjusted linear regression models also showed similar results.

**Conclusions:**

Teenagers who spent more time outdoors in a swimsuit/direct sunlight or got more sunburns tended to use indoor tanning more frequently. These findings provide evidence that teenagers with stronger sun-seeking behaviors may have more exposure to artificial ultraviolet radiation as well.

## Key points

Question: How are outdoor sun-seeking behaviors associated with indoor tanning behavior?

Findings: This large study using data from the Nurses’ Health Study II (NHSII) found that females who spent more time outdoors or got more sunburns tended to use indoor tanning more frequently than those who rarely or never engaged in outdoor sun-seeking behaviors.

Meaning: The findings reveal the associations between outdoor sun-seeking behaviors and indoor tanning behavior, which could increase awareness of UV-seeking behaviors and underscore appropriate interventions directed at adolescent and young adult females with such behaviors.

## Introduction

Skin cancer is the most common malignancy in the United States [1]. Specifically, melanoma, a malignant form of skin cancer, is one of the most common cancers among young people [2]. Ultraviolet (UV) radiation is the major etiologic agent in skin cancer development and in 2009 was classified as “carcinogenic to humans” (group1) by the International Agency for Research on Cancer [3]. Repeated sunburns as an inflammatory reaction to UV radiation damage to the skin’s outermost layers [4], raise the risk of skin cancer by altering a tumor-suppressing gene [5]. Tanning through extended outdoor sun exposure or use of indoor tanning beds is defined as intentional exposure to UV radiation that darkens the skin for cosmetic purposes [6, 7].

Despite risks associated with tanning, young people are likely to have greater sun exposure and a strong desire to tan but are less likely to be concerned about sun protection [8–12]. This is probably due to the reinforcing effects of UV radiation created by the many neuropeptides released from the skin after exposure to UV radiation, which can offer relaxation and increase the sense of well-being [13, 14]. In fact, dopaminergic efflux is correlated with tanning severity [15]. In addition, indoor tanning is more popular among US adolescent females than adolescent males [16]. Also, non-Hispanic White adolescent frequent sunbathers were more likely to be females and far more likely to use indoor tanning beds than those who never sunbathed [17]. Moreover, at early ages, exposure to UV has more harmful and longer effects on skin cancer risk than later in life [18]. Therefore, understanding tanning behaviors during early life is important for skin cancer prevention among young US females through guiding potential interventions for control of sun-seeking behaviors and indoor tanning use in high-risk groups.

Among American high school students, tanning has declined in recent years, but has not stopped entirely [16]. For example, 8.4% of US white female high school students tanned indoors in 2019 [19, 20]; about a quarter of those who reported any indoor tanning did so ≥ 25 times in 2018 [21]. Accordingly, female-focused larger investigations of the association between outdoor and indoor tanning behaviors at early ages are warranted.

Given the above findings, we hypothesized that females who had stronger sun-seeking behaviors would tend to use indoor tanning facilities more frequently. Thus, we sought to investigate the association between a series of outdoor sun-seeking behaviors and frequency of indoor tanning using data from the Nurses’ Health Study II (NHS II), a large well-characterized cohort of US females.

## Methods

### Study population

The NHS II is a large prospective cohort focusing on health and disease risk factors. In 1989, the cohort enrolled 116,429 female nurses aged 25 to 42 years who resided in one of 14 US states with a large number of registered nurses. The initial self-administered questionnaires asked questions regarding demographic factors, medical/familial histories, and early-life health-related information. Some health habits at early ages were additionally asked in follow-up surveys. Details of this cohort have been described previously [22]. In this study, we included white females whose answers included the average annual frequency of indoor tanning during high school/college. The study protocol was approved by the institutional review boards of the Brigham and Women’s Hospital and Harvard T.H. Chan School of Public Health, and those of participating registries as required. Written informed consent was also obtained from all study participants. This study followed the Strengthening the Reporting of Observational Studies in Epidemiology (STROBE) reporting guidelines.

### Outcome

The questionnaire asked participants to report their frequency of tanning bed use during high school/college by choosing from the following 6 categories: none, 1–2, 3–5, 6–11, 12–23, and ≥ 24 times/year. We recategorized the average annual indoor tanning frequencies into 4 categories (none, 1–2, 3–11, and ≥ 12 times/year) [23]. We also converted the responses from the original 6 categories to continuous values (median of each category) by assuming that each level of the categories is 0, 1.5, 4.5, 9, 18, and 24 times per year, respectively, to test the associations not only in logistic regression models but also in linear regression models to validate the results and remedy the shortcomings of logistic regression models, such as arbitrary cutoffs and adjusted odds ratios (aORs) [24, 25].

### Exposures

We collected participants’ responses to the outdoor sun-seeking behavior-related questions, including number of times/week outdoors in a swimming suit as a teenager (5 categories: <1, 1, 2, several, and daily), percentage of time wearing sunscreen at the pool/beach as a teenager (5 categories: 100%, 75%, 50%, 25%, and 0%), weekly hours spent outdoors in direct sunlight during high school/college (3 categories: <1, 2–4, and ≥ 5 h/week), and number of severe sunburns that blistered between ages 15 and 20 (5 categories: never, 1–2, 3–4, 5–9, and ≥ 10).

### Covariates

We included the following participant-reported variables as our covariates: age, average number of cigarettes per day during ages 15–24, number of drinks of alcohol during ages 15–22, average frequency of strenuous physical activity/sports (i.e., swimming, aerobics, field hockey, basketball, cycling, and running) at least twice per week during high school and ages 18–22, hair color (red, blonde, light brown, dark brown, or black), family history of melanoma (yes vs. no), personal history of major chronic diseases (yes vs. no) [26], and number of moles on lower legs (none, 1–2, 3–9, and ≥ 10). We defined a participant as having had major chronic diseases when they reported any cancer, myocardial infarction, stroke, type 2 diabetes, hypertension, inflammatory bowel disease, rheumatoid arthritis, and/or multiple sclerosis in their medical history.

### Statistical analysis

We calculated age-standardized characteristics and outdoor sun-seeking behaviors according to average annual frequency of indoor tanning bed usage during high school/college. Continuous variables were presented as mean (standard deviation, SD) and categorical variables were presented as percentage. We performed age and multivariable-adjusted multinomial logistic regression using the 4 categorized outcomes to calculate aORs with 95% confidence intervals (CIs) for the association between outdoor sun-seeking behaviors and the average annual frequency of indoor tanning bed use during early life. In the multivariable-adjusted model, for each exposure variable, we adjusted for all covariates described above. In the same way, we conducted age- and multivariable-adjusted linear regression but using the continuous outcome values, yielding adjusted *β* (95% CI). In all regression models, to reduce the number of observations deleted because of missing values in covariates, we created indicator variables of the missing data in all covariates and adjusted them as well.

All statistical tests were two-sided, and we determined statistical significance using a threshold of p-value < 0.05. All analyses were carried out using Statistical Analysis System software (version 9.4; SAS Institute, Cary, NC).

## Results

Age-standardized characteristics of participants according to frequencies of tanning bed usage during high school/college are shown in Table 1. A total of 81,746 eligible female nurses were included in the study; their ages when they responded to the exposures/outcome items were early 30s on the average. 7,415 (9.1%) used indoor tanning beds during high school/college, among whom 1,227 (1.5%) reported that they used indoor tanning beds with high frequency (≥ 12 times/year). Females who used tanning beds more often during early life tended to be younger and were heavier smokers and drinkers during their early life. They also tended to engage in more strenuous physical activity or sports during their early life and had higher nevus counts on their lower legs. The distributions of other characteristics, such as hair color, family history of melanoma, and history of major chronic diseases, were similar across the 4 categories of the average frequency of indoor tanning bed usage.

**Table 1:** Age-standardized characteristics according to average annual frequency of indoor tanning bed usage during high school/college in US females (*N* = 81,746) ^a^

Characteristics	None	1–2	3–11	≥ 12
**Number of participants**	74,331	3,400	2,788	1,227
**Age, years**	34.8 (4.6)	32.8 (5.0)	32.2 (5.0)	31.3 (4.8)
**Number of cigarettes per day ages 15–24**	11.1 (7.6)	10.9 (8.1)	11.1 (8.4)	12.1 (8.6)
**Usual number of alcoholic drinks ages 15–22** ^**b**^	1.8 (2.9)	2.1 (2.9)	2.3 (3.2)	2.5 (3.5)
**Frequency of strenuous physical activity/sports at least twice per week ages 14–22** ^**c**^	4.7 (3.6)	5.0 (3.6)	5.1 (3.6)	5.2 (3.6)
**Hair color, %**				
Red	3.6	2.7	3.5	1.9
Blonde	14.6	16.1	16.1	17.8
Dark brown	35.7	34.1	36.2	32.8
Black	3.1	1.2	1.3	0.8
**Family history of melanoma, %**	4.2	4.8	6.5	5.0
**History of major chronic diseases, %** ^**d**^	12.3	12.7	10.9	10.6
**Number of moles on lower legs, %**				
1–2	18.5	18.4	19.6	18.0
3–9	16.6	17.3	18.4	17.0
10+	14.1	16.9	15.2	19.7
**The number of times per week spent outdoors in a swimsuit as a teenager, %**				
<1	14	7.9	6.6	5.6
1	9.5	8.3	7.3	4.8
2	16.0	14.9	14.0	10.6
Several	45.1	50.9	51.7	53.9
Daily	15.4	18.0	20.4	25.1
**Percentage of time wearing sunscreen at the pool/beach as a teenager**				
100%	1.4	0.9	0.7	1.5
75%	4.1	3.7	3.1	3.1
50%	9.9	9.7	9.4	7.9
25%	22.4	26.2	23.7	20.5
0%	62.2	59.5	63.1	67.0
**Weekly hours spent outdoors in direct sunlight during the day during high school/college, %**				
<1	7.5	4.7	3.2	3.2
2–4	32.9	31.1	24.0	19.6
5+	59.6	64.2	72.8	77.2
**Number of severe sunburns that blistered between ages 15–20, %**				
Never	35.5	25.5	27.9	26.1
1–2	38.3	42.6	40.4	39.0
3–4	16.6	20.2	18.4	18.3
5–9	7.2	8.6	9.7	11.0
10+	2.4	3.1	3.6	5.6

*Note*: (a) Values are means (SD) for continuous variables, percentages for categorical variables, and standardized to the age distribution of the study population except for baseline age; (b) Number of usual drinks equals total of bottles/cans of beer, 4 oz. glasses of wine, or shots of liquor; (c) Strenuous (aerobic) physical activity/sports includes swimming, aerobics, field hockey, basketball, cycling, and running; (d) Major chronic diseases include cancer, myocardial infarction, stroke, type 2 diabetes, hypertension, inflammatory bowel disease, rheumatoid arthritis, and multiple sclerosis

The age- and multivariable-adjusted odds ratios for the association between outdoor sun-seeking behaviors and indoor tanning frequency during high school or college are presented in Table 2. In multivariable-adjusted models, being outdoors more often in a swimsuit as a teenager, spending more time in direct sunlight during the day during high school/college, and experiencing more frequent severe sunburns between ages 15 and 20 was significantly associated with higher frequency of average annual indoor tanning bed usage. Specifically, teenagers who spent 7 times/week outdoors in a swimsuit (aOR, 95% CI for daily vs. <1 per week: 2.68, 1.76–4.09) or who had ≥ 10 sunburns that blistered (aOR, 95% CI for ≥ 10 vs. never: 2.18, 1.53–3.10) were more likely to use indoor tanning beds **≥** 12 times/year. Also, teenagers/undergraduates who spent ≥ 5 h per week in direct sunlight were more likely to use indoor tanning **≥** 12 times/year (aOR, 95% CI: 2.18, 1.39–3.44) than those who spent < 1 h per week in direct sunlight. However, there was not a significant association between sunscreen usage at the pool/beach and the frequency of indoor tanning bed usage.

**Table 2:** Age and multivariable-adjusted logistic regression for associations of sun-seeking behaviors with average annual frequency of indoor tanning bed usage during high school/college

	None	1–2			3–11			≥ 12			
	N ^a^	N ^a^	Unadjusted Model	Adjusted Model ^b^	N ^a^	Unadjusted Model	Adjusted Model ^b^	N ^a^	Unadjusted Model		Adjusted Model ^b^
**Number of times per week spent outdoors in a swimsuit as a teenager**											
<1	9,270	215	1.00	1.00	140	1.00	1.00	54	1.00	1.00	
1	6,323	230	1.49 (1.23–1.79)^*^	1.31 (1.00-1.71)	164	1.60 (1.27–2.01) ^*^	1.45 (1.06–1.98) ^*^	51	1.25 (0.85–1.84)	1.34 (0.79–2.27)	
2	10,603	444	1.65 (1.40–1.95) ^*^	1.50 (1.19–1.89) ^*^	331	1.84 (1.51–2.25) ^*^	1.44 (1.09–1.91) ^*^	110	1.52 (1.10–2.11) ^*^	1.22 (0.76–1.96)	
Several	29,759	1,553	1.99 (1.72–2.30) ^*^	1.69 (1.38–2.07) ^*^	1,304	2.48 (2.08–2.96) ^*^	2.01 (1.58–2.55) ^*^	578	2.70 (2.04–3.58) ^*^	2.45 (1.65–3.64) ^*^	
Daily	10,133	559	2.07 (1.77–2.44) ^*^	1.80 (1.43–2.26) ^*^	523	2.87 (2.37–3.47) ^*^	2.25 (1.73–2.93) ^*^	276	3.71 (2.76–4.98) ^*^	2.68 (1.76–4.09) ^*^	
**Percentage of time wearing sunscreen at the pool or beach as a teenager**											
100	950	34	1.00	1.00	20	1.00	1.00	18	1.00	1.00	
75	2,575	127	1.29 (0.88–1.90)	1.16 (0.68–1.96)	92	1.56 (0.95–2.55)	2.23 (1.05–4.76) ^*^	44	0.81 (0.46–1.41)	0.63 (0.29–1.38)	
50	6,296	317	1.34 (0.93–1.92)	1.09 (0.67–1.78)	270	1.91 (1.20–3.02) ^*^	2.39 (1.16–4.92) ^*^	101	0.78 (0.47–1.30)	0.66 (0.33–1.33)	
25	14,310	871	1.67 (1.18–2.37) ^*^	1.42 (0.89–2.27)	660	2.14 (1.37–3.37) ^*^	2.43 (1.19–4.95) ^*^	266	0.96 (0.59–1.56)	0.88 (0.46–1.70)	
0	40,614	1,646	1.31 (0.93–1.85)	1.07 (0.67–1.71)	1,412	2.03 (1.30–3.17) ^*^	2.29 (1.13–4.64) ^*^	635	1.11 (0.69–1.78)	0.88 (0.46–1.69)	
**Weekly hours spent outdoors in direct sunlight during the day during high school/college**											
<1	5,574	142	1.00	1.00	76	1.00	1.00	33	1.00	1.00	
2–4	24,162	1,013	1.53 (1.28–1.83) ^*^	1.59 (1.23–2.06) ^*^	655	1.80 (1.42–2.29) ^*^	1.86 (1.31–2.65) ^*^	222	1.36 (0.94–1.96)	1.12 (0.70–1.81)	
≥5	43,564	2,221	1.76 (1.48–2.09) ^*^	1.59 (1.24–2.04) ^*^	2,033	2.89 (2.29–3.64) ^*^	2.83 (2.01–3.98) ^*^	958	2.96 (2.09–4.20) ^*^	2.18 (1.39–3.44) ^*^	
**Number of severe sunburns that blistered between ages 15–20**											
Never	26,144	911	1.00	1.00	784	1.00	1.00	341	1.00	1.00	
1–2	28,317	1,444	1.45 (1.33–1.58) ^*^	1.42 (1.25–1.62) ^*^	1,161	1.35 (1.23–1.48) ^*^	1.24 (1.08–1.42) ^*^	490	1.31 (1.14–1.50) ^*^	1.03 (0.84–1.26)	
3–4	12,289	656	1.56 (1.41–1.73) ^*^	1.57 (1.35–1.82) ^*^	492	1.36 (1.22–1.53) ^*^	1.27 (1.08–1.51) ^*^	211	1.36 (1.14–1.61) ^*^	1.10 (0.87–1.41)	
5–9	5,374	280	1.55 (1.35–1.77) ^*^	1.49 (1.22–1.81) ^*^	251	1.63 (1.41–1.88) ^*^	1.48 (1.20–1.82) ^*^	119	1.81 (1.46–2.23) ^*^	1.39 (1.04–1.87) ^*^	
≥10	1,803	98	1.61 (1.30-2.00) ^*^	1.64 (1.24–2.15) ^*^	92	1.78 (1.43–2.23) ^*^	1.72 (1.30–2.29) ^*^	62	2.82 (2.14–3.72) ^*^	2.18 (1.53–3.10) ^*^	

*Note*: (a) Number of participants; (b) Adjusted for age (continuous), number of cigarettes per day ages 15–24 (continuous), number of alcoholic drinks ages 15–22 (continuous), frequency of strenuous physical activity/sports at least twice per week at ages 14–22 (continuous), hair color (red, blonde, light brown, dark brown, or black), family history of melanoma (yes, no), personal history of major chronic diseases (yes, no), and number of moles on lower legs (none, 1–2, 3–9, 10+); * *P* < 0.05

Table 3 shows the linear regression results on the association between outdoor sun-seeking behaviors and average annual frequency of indoor tanning bed use. Overall, the significant associations we observed in the logistic regression were also seen in the linear models. Teenagers who spent time outdoors in a swimsuit every day (adjusted *β*, 95% CI for daily vs. <1 per week: 0.25, 0.16–0.34) or who had ≥ 10 sunburns that blistered (adjusted *β*, 95% CI for ≥ 10 vs. never: 0.45, 0.29–0.62) were more likely to use indoor tanning beds. Also, teenagers/undergraduates who spent ≥ 5 h per week in direct sunlight used indoor tanning more (adjusted *β*, 95% CI: 0.29, 0.18–0.40) than those who spent < 1 h per week in direct sunlight.

**Table 3:** Age and multivariable-adjusted linear regression for associations of sun-seeking behaviors with average annual frequency of indoor tanning bed usage during high school/college

	N ^a^	Unadjusted Model	Adjusted Model ^b^
**Weekly time spent outdoors in a swimsuit as a teenager**			
<1	9,679	1.00	1.00
1	6,768	0.07 (-0.01-0.16)	0.05 (-0.07-0.17)
2	11,488	0.13 (0.05–0.20)^*^	0.03 (-0.08-0.13)
Several	33,194	0.50 (0.42–0.57)^*^	0.32 (0.21–0.43)^*^
Daily	11,491	0.32 (0.26–0.38)^*^	0.25 (0.16–0.34)^*^
**Percentage of time wearing sunscreen at the pool or beach as a teenager**			
100	1,022	1.00	1.00
75	2,838	-0.002 (-0.20-0.19)	-0.07 (-0.35-0.21)
50	6,984	0.03 (-0.15-0.21)	-0.02 (-0.28-0.23)
25	16,107	0.12 (-0.05-0.30)	0.08 (-0.16-0.33)
0	44,307	0.13 (-0.04-0.30)	0.03 (-0.21-0.27)
**Weekly hours spent outdoors in direct sunlight during the day during high school/college**			
<1	5,825	1.00	1.00
2–4	26,052	0.09 (0.01–0.17)^*^	0.07 (-0.04-0.18)
≥5	48,776	0.39 (0.31–0.46)^*^	0.29 (0.18–0.40)^*^
**Number of severe sunburns that blistered between ages 15–20**			
Never	28,180	1.00	1.00
1–2	31,412	0.13 (0.09–0.18)^*^	0.01 (-0.05-0.08)
3–4	13,648	0.15 (0.10–0.21)^*^	0.06 (-0.02-0.15)
5–9	6,024	0.29 (0.21–0.37)^*^	0.17 (0.06–0.28)^*^
≥10	2,055	0.53 (0.41–0.66)^*^	0.45 (0.29–0.62)^*^

*Note*: (a) Number of participants; (b) Adjusted for age (continuous), number of cigarettes per day at ages 15–24 (continuous), number of alcoholic drinks at ages 15–22 (continuous), frequency of strenuous physical activity/sports at least twice per week at ages 14–22 (continuous), hair color (red, blonde, light brown, dark brown, or black), family history of melanoma (yes, no), personal history of major chronic diseases (yes, no), and number of moles on lower legs (none, 1–2, 3–9, 10+); * *P* < 0.05

## Discussion

Here we investigated the association between outdoor sun-seeking behaviors and average annual frequency of indoor tanning during early life using survey data from the NHS II. We found that females who spent more time outdoors in a swimsuit or in direct sunlight during the day, or who experienced more frequent severe sunburns, were higher-frequency indoor tanners during their early life.

The significant association between outdoor sun-seeking behaviors and indoor tanning behavior should cause concern, because excessive exposure to UV radiation is possible [17], and such excessive exposure can increase health risks, e.g., skin atrophy and malignancy [27]. Furthermore, it is well known that such exposure is linked to the three most common types of skin cancer, basal cell carcinoma, squamous cell carcinoma, and malignant melanoma [28]. Despite the risks, exposure to UV radiation is related to central reward mechanisms [29]. To be specific, exposure to UV radiation, triggering DNA damage to the skin, activates the p53 protein to induce production of pro-opiomelanocortin (POMC) and its derivative *β*-endorphin [30]. *β*-endorphin, the most abundant endogenous opioid, acts on central neural dopamine receptors to make the tanner relaxed and increase her sense of well-being [13, 31]. Moreover, frequent tanning may chronically elevate tanners’ endorphin levels, stimulating the addictive effects of UV exposure [32].

We observed significant associations between the time spent outdoors in a swimsuit or in direct sunlight in daytime as a teenager and indoor tanning bed use during high school/college. Weekly frequency of spending time outdoors wearing a swimsuit or in direct sunlight reflect almost the same participant characteristics (an individual’s sun-seeking tendency), and high frequencies of both may raise the risk of skin cancer via increased exposure to UV rays. This can also strengthen stimulation of the reward system and further increase the sun-seeking tendency [33]. In turn, increased sun-seeking may also lead to increased use of indoor tanning facilities, which emit UV light that is far more intense than natural sunlight, initiating a vicious cycle [17]. Thus, education should be provided to teenagers with strong sun-seeking behaviors to discourage them from seeking long exposure to direct sunlight during the day or to artificial UV light. Furthermore, because teenagers’ behavior is likely to be influenced by others because of the psychological pressure to conform [34, 35], school health stakeholders and parents should encourage teenagers to practice sun safety.

We found the strongest positive associations between undergoing ≥ 10 severe sunburns that blistered between ages 15 and 20 and usage of indoor tanning beds of ≥ 12 times. Such a relationship was also observed in other studies reporting positive associations between indoor tanning and sunburns because of tan-seeking behavior [36, 37]. Five or more sunburns increase the risk of development of melanoma [38]. Indoor tanning bed use is also an established risk factor for melanoma [39, 40]. To mitigate risk, dermatologists might recommend published consensus guidelines for indoor tanning usage among teenagers with more frequent severe sunburns, and public health officers or school health teachers could provide preventive education to help protect teenagers from severe sunburns, such as encouraging the use of sunscreen outdoors.

Sunscreens are an important source of protection against UV exposure and can effectively reduce the incidence of skin cancers [41]. However, our study did not demonstrate the inverse association between the percentage of time wearing sunscreen at the pool/beach and the use of indoor tanning beds, which differs from previous findings [34, 42, 43]. A study among US high school students found that white female students who reported always using sunscreen were significantly less likely to use indoor tanning equipment [42]. Another study pointed out that non-use of sunscreen at the pool/beach was an independent predictor of indoor tanning use [34]. Moreover, a study based on US 2015 National Health Interview Survey data also found that those who frequently tanned indoors were more likely to rarely/never use sunscreen [43]. Inconvenience and the lack of perceived need to apply sunscreen are known as the major reasons for not using sunscreen [44]. However, indoor tanning-seeking behavior may be more likely to be due to needs for UV exposure, regardless of how the individual perceives the importance of sunscreen, which may partially explain why we observed no such association.

Many countries have recently moved to ban those younger than 18 from using indoor tanning beds [45–47]. However, adults who use tanning beds also deserve special attention, especially those under age 25. Our data showed that people who used tanning beds more often during early life tended to be younger, and also had more moles on their lower legs, which was consistent with the results of a previous study [23]. As shown in Table 1, heavier smokers as well as drinkers tended to use tanning beds more often. As the reward mechanisms affected by smoking are similar to those associated with indoor tanning bed use [48], those interactions deserve further study. Females who engaged in more strenuous physical activity or sports sought to use indoor tanning beds more, which might be related to a need to relax and relieve pain. Persistent indoor tanning is positively associated with both risk taking [49] and unhealthy lifestyle [17] behaviors. Therefore, those results suggest that teenagers with a cluster of such unhealthy behaviors should be considered high-risk groups and receive closer attention.

Our study has several strengths, including a relatively large sample size (*N* = 81,746) compared to the previous studies with similar research topics. In addition, detailed collection of baseline information, lifestyle data, and medical history using well-designed and validated self-reported questionnaires allowed us to adjust for widely recognized potential confounders in the relationship of interest and to develop valid reference for the association. Moreover, we used longitudinal exposure/outcome data, which may be more valuable than records during shorter period in measuring habit of the participants that affected their indoor tanning facilities usage during early life.

Our study also has several limitations. First, the information we collected on study variables may introduce recall bias; however, there was only a slim chance that participants would exaggerate or understate their actual habit/values, because the cohort study design meant that when they reported, they were not under any pressure to do so. Second, residual and unmeasured confounding cannot be fully ruled out because of the nature of the survey data; however, we adjusted for many well-known confounding factors. Third, though some misclassification is inherent from self-reported questionnaires, the questionnaire has been extensively validated in subsamples of this cohort, and any misclassification would likely be some nondifferential error to bias our results toward the null. Fourth, the sunscreen usage item does not include information on which sun protection factor was applied. Fifth, we have converted the categories of exposures to continuous values using each category’s median value for the linear regression, which may lead to biased results. Lastly, we studied only compliant healthy White professional female nurses belonging to a specific social stratum, which may create a lack of external validity.

In conclusion, our study reveals associations between outdoor sun-seeking behaviors and average annual frequency of indoor tanning bed usage, suggesting that young females who spent more time outdoors and had more frequent severe sunburns tended to use indoor tanning more frequently than those with fewer sun-seeking behaviors. If properly publicized, these findings could increase awareness of needs for UV exposure and the unmet needs for systematic and continuous interventions regarding access to indoor tanning beds among female adolescents and college students with strong outdoor sun-seeking behaviors. This work may contribute vital public health messaging for young US female.

## Data Availability

The data that support the findings of this study are available from Brigham and Women’s Hospital and Harvard T.H. Chan School of Public Health, but restrictions apply to the availability of these data, which were used under license for the current study, and so are not publicly available. However, data described in the manuscript, code book, and analytic code are available from the authors upon reasonable request to corresponding author and with permission of Brigham and Women’s Hospital and Harvard T.H. Chan School of Public Health.
